# Reviewer Acknowledgement 2012

**DOI:** 10.1186/1755-7682-6-9

**Published:** 2013-04-05

**Authors:** Manuel Menéndez-González

**Affiliations:** 1 Hospital Álvarez-Buylla, Universidad de Oviedo, Oviedo, Spain

## Abstract

**Contributing reviewers:**

The Editor-in-Chief of *International Archives of Medicine* would like to thank all our reviewers who have contributed to the journal in Volume 5 (2012).

## 

Khalid Abidi

Morocco

Ishtiaque Ahmaed

United States of America

Moin Arain

Pakistan

Enayatollah Bakhshi

Iran

Jihane Belayachi

Morocco

Henning Budde

Iceland

Tatiana Carvalho

Brazil

Beverley Copnell

Australia

João Correa

Brazil

Helen Davies

United Kingdom

Jerome Delotte

France

Olufemi Fasanmade

Nigeria

Geoff Frawley

Australia

Ana Clara Cr Gonçalves

Brazil

Jan Willem Gorter

Canada

Uwe Gross

United Kingdom

Buddika Gurunayaka

Sri Lanka

Fitsum Habte

Ethiopia

Sumedh Hoskote

United States of America

Gaetano Iapichino

Italy

Tsutomu Inaoka

Japan

Ghassan Jamaleddine

United States of America

Rohitha Jayamaha

Sri Lanka

Panagiotis Kafas

Greece

Makarand Khochikar

India

Danai Kitkungvan

United States of America

Hildtraud Knopf

Germany

Babatope Kolawole

Nigeria

Brian Koo

United States of America

Matthias Kreppel

Germany

Lucas Lima Ferreira

Brazil

Angela Lombardi

Italy

Heraldo Lorena Guida

Brazil

Fernando Luiz Affonso Fonseca

Brazil

Paulo Marchetti

Brazil

Franciele Marques Vanderlei

Brazil

Miguel Mendez-Rojas

Mexico

Alexandra Meyer

Germany

Isadora Lessa Moreno

Brazil

Giuseppe Murdaca

Italy

Germano Mwabu

Kenya

Isabella Nascimento

Brazil

Thusha Nawasiwatte

Sri Lanka

Bruno Nazar

Brazil

Bennett Nwanguma

Nigeria

Madalina Opreanu

United States of America

Raffaele Pascarella

Italy

Gene Pesola

United States of America

Razine Rachid

Morocco

William Roberts

United States of America

Renata Rossi

Brazil

Mohamed Salama

Egypt

Irene Savelieva

United Kingdom

Abhishek Singh

India

Andrzej Szuba

Poland

Carlos Tavares

Cape Verde

Marco Andre Urbach Mezzasalma

Brazil

Henk R van Buuren

Netherlands

Luiz Carlos Marques Vanderlei

Brazil

Carlos Vazquez

Spain

Ian Wu

United States of America

Valdelias Xavier Pereira

Brazil

Ti-Fei Yuan

China

